# Virtual 2-D map of the fungal proteome

**DOI:** 10.1038/s41598-021-86201-6

**Published:** 2021-03-23

**Authors:** Tapan Kumar Mohanta, Awdhesh Kumar Mishra, Adil Khan, Abeer Hashem, Elsayed Fathi Abd-Allah, Ahmed Al-Harrasi

**Affiliations:** 1 Natural and Medical Sciences Research Center, University of Nizwa, Nizwa, 616 Oman; 2 Department of Biotechnology, Yeungnam University, Gyeongsan, Gyeongsangbuk-do 38541 Republic of Korea; 3 Botany and Microbiology Department, College of Science, King Saud University, P.O. Box. 2460, Riyadh, 11451 Saudi Arabia; 4 Mycology and Plant Disease Survey Department, Plant Pathology Research Institute, ARC, Giza, 12511 Egypt; 5 Plant Production Department, College of Food and Agricultural Sciences, King Saud University, P.O. Box. 2460, Riyadh, 11451 Saudi Arabia

**Keywords:** Computational biology and bioinformatics, Microbiology, Molecular biology, Systems biology, Biochemistry, Proteins, Proteomics

## Abstract

The molecular weight and isoelectric point (*pI*) of the proteins plays important role in the cell. Depending upon the shape, size, and charge, protein provides its functional role in different parts of the cell. Therefore, understanding to the knowledge of their molecular weight and charges is (*pI*) is very important. Therefore, we conducted a proteome-wide analysis of protein sequences of 689 fungal species (7.15 million protein sequences) and construct a virtual 2-D map of the fungal proteome. The analysis of the constructed map revealed the presence of a bimodal distribution of fungal proteomes. The molecular mass of individual fungal proteins ranged from 0.202 to 2546.166 kDa and the predicted isoelectric point (*pI*) ranged from 1.85 to 13.759 while average molecular weight of fungal proteome was 50.98 kDa. A non-ribosomal peptide synthase (RFU80400.1) found in *Trichoderma arundinaceum* was identified as the largest protein in the fungal kingdom. The collective fungal proteome is dominated by the presence of acidic rather than basic *pI* proteins and Leu is the most abundant amino acid while Cys is the least abundant amino acid. *Aspergillus ustus* encodes the highest percentage (76.62%) of acidic *pI* proteins while *Nosema ceranae* was found to encode the highest percentage (66.15%) of basic *pI* proteins. Selenocysteine and pyrrolysine amino acids were not found in any of the analysed fungal proteomes. Although the molecular weight and *pI* of the protein are of enormous important to understand their functional roles, the amino acid compositions of the fungal protein will enable us to understand the synonymous codon usage in the fungal kingdom. The small peptides identified during the study can provide additional biotechnological implication.

## Introduction

Genomic studies have contributed enormously towards understanding the molecular mechanisms underlying cellular development through the expression of proteins, collectively referred to as the proteome^1,2^. Proteins play a qualitative and quantitative role in growth, development, and stress tolerance of an organism^3–6^. They are the structural and functional units of a cell comprising a chain of amino acids which are arranged as determined by their coding sequences (mRNA)^7–9^. Different proteins are synthesized based on the immediate and developmental needs required by the cell as part of programmed development or specific response signals^10,11^. Proteins often undergo post-translational modification and are frequently targeted for delivery to specific sub-cellular locations^12,13^. The function of a protein depends upon its molecular structure and is also affected by the post-translational modifications that occur^14–17^. Additionally, the sub-cellular localization of a protein plays a critical role in defining its function since different cellular compartments regulate various physiological and biochemical functions^18–20^. Similarly, post-translational modifications also play multifunctional regulatory roles that change the function of a protein and regulate biological processes^21–23^. The *pH* of the cytoplasm plays an important role for the post-translational modification that occur and directing the sub-cellular localization of proteins^24–27^. Tight regulation of proton fluxes and the maintenance of a *pH* gradient across cellular membranes play critical roles in cellular homeostasis^24,28–30^. Different proteins respond to diverse *pH* gradients across cellular compartments that regulate cell metabolism^31,32^. Large deviations in *pH*, however, need to be avoided to maintain normal metabolic and intracellular biochemical processes. The isoelectric point (*pI*) of a protein molecule plays a major role in its contribution to cellular homeostasis. The *pI* is the *pH* at which the protein molecule carries no electrical charges. The net charge of a protein molecule is affected by the *pH* gradient of a cell and by sub-cellular compartments. This can lead to a more positive or negatively charged protein molecule and determine the gain or loss of protons (H^+^).

Although protein synthesis occurs in the cytoplasm (*pH* 7.4–7.7)^33^, proteins are often transported to different sub-cellular locations, including the endoplasmic reticulum, Golgi complexes, mitochondria, vacuoles (*pH* 6.2), cell membranes, lysosomes, and other compartments after synthesis and processing^34^. Therefore, different protein needs to undergo modifications that are appropriate to the *pH* gradient that exists across different cellular compartments. The *pH* of the cytoplasm is slightly alkaline^35,36^ while the *pH* of sub-cellular organelles, including the vacuole and lysosomes, are slightly acidic^34^. The *pI* of a protein has been used as a standard parameter to distinguish proteins^37^. The molecular mass of a protein also plays a vital role in its translocation across the cellular compartments^38,39^. Therefore, knowing the *pI* and molecular mass of a protein provides critical information that can help to determine its functional role in a cell. The advent of next-generation, high-throughput DNA sequencing allowed for the generation of complete genome sequences of more than one thousand fungal species. In the current study, the annotated protein sequences of 689 species derived from completed genome sequencing efforts within public repositories were used to construct a collective 2-D map of the fungal proteome.

## Results and discussion

### Size of the fungal genome is not directly proportional to the number of amino acids

A proteome-wide study of 689 fungal species was conducted to characterize the molecular mass, isoelectric point, and amino acid composition of the fungal kingdom. The study comprised 7.159 million protein sequences from ten fungal groups, Ascomycota (466 species), Basidiomycota (148 species), Blastocladiomycota (1 species), Chytridiomycota (8 species), Glomeromycota (1 species), Microsporidia (26 species), Mucoromycota (23 species), Neocallimastigomycota (1 species), Opisthokonta (1 species), and Zoopagomycota (14 species). Results indicated that, *Fibula rzhizoctina* (Basidiomycota), commonly known as the cuckoo fungus, encoded the highest number protein sequences, totalling 32,854 (Supplementary File 1). The largest genome size in the fungal kingdom was found in *Neocallimastix californiae* (193.032 Mb) followed by *Tuber magnatum* (192.781 Mb) (Supplementary File 2). Although *Neocallimastix californiae* (193.032 Mb) had the largest genome, *Fibula rhizoctonia* (genome size 95.118 Mb) encoded a higher number of amino acids (10.940068 million) (Supplementary Fig. 1A, Supplementary File 1). The genome size of all 689 fungal species was compared with the number of amino acids using regression analysis to determine the correlation between genome size and the number of amino acids in fungi (Supplementary Fig. 2A,B). Results indicated that genome size in the fungal kingdom is not proportional to the number of amino acids encoded by a genome (Supplementary Fig. 2A). This indicates that the larger fungal genomes contain a greater number of introns and other non-coding nucleotide sequences in their genomes. A linear regression analysis of the presence of a specific amino acid number in a fungal species revealed that Met (0.993) had the highest correlation coefficient, followed by Thr (0.9917), Leu (0.9913), Trp (0.9907), and Val (0.9906). The lowest correlation coefficient was found for Asn (0.8828) (Supplementary Fig. 2B).

### The average molecular mass of fungal protein was 50.96 kDa and fungal proteome was 518539.109524477 kDa

The average molecular mass of fungal proteins was 50.96 kDa (Supplementary File 3). The highest average molecular mass of proteins was found in *Aspergillus ustus* (70.7 kDa), followed by *Ascosphaera apis* (69.13 kDa), *Candida boidinii* (66.71 kDa), *Moesziomyces antacticus* (66.37 kDa), and *Anthracocystis floculosa* (66.21 kDa) (Supplementary File 3). The lowest average molecular mass of proteins found in *Moniliopthora perniciosa* (20.34 kDa), *Aspergillus wentii* (26.94), and *Hepatospora eriocheir* (26.94 kDa) (Supplementary File 3). Approximately 9.29% of fungal proteins have a mass > 100 kDa. When the molecular mass of the whole proteome of individual species was analysed, *Rhizophgus clarus* was found to have the heaviest proteome with a total molecular mass of 1,210,411.214 kDa, followed by *Fibularhizoctina* sp. (1,204,483.772 kDa), *Diversispora versiformis* (1,171,535.352 kDa), and *Neocallimatrix californiae* (1,120,727.893 kDa). The average molecular mass of a fungal proteome was found to be 518,539.109 kDa (Supplementary File 3). Further analysis revealed that the largest protein in the fungal kingdom was a non-ribosomal peptide synthase (accession number RFU80400.1) found in *Trichoderma arundinaceum*. This protein has a molecular mass of 2546.166 kDa and is the first fungal protein ever reported with highest molecular mass. Other high molecular mass proteins include an amino acid adenylation protein (2542.314 kDa, 23,089 aa, accession number PTB76898.1) in *Trichoderma longibrachiatum*, NRPS protein (2527.788 kDa, 22,960 aa, accession number OTA01063.1) in *Trichoderma parareesei*, and a non-ribosomal peptide synthase (2314.108 kDa, 20,891 aa, accession number EHK18913.1) in *Trichoderma virens*. The largest protein molecules are act as molecular machines of the cell that provides the structural and functional unit to the cell^40^. Non-ribosomal peptide synthase is a multi-domain protein that produces several important secondary metabolites for its virulence^41^. Fungal NRPS protein comprises of several pharmacological relevant compounds including β-lactam anti-biotics, echinocandin antibiotics, and cyclosporins^41^.

The molecular mass of the smallest fungal protein/peptide was 0.202 kDa (hypothetical protein, accession number KFA68322.1) found in *Stachybotrys chlorohalonata*. It is a dipeptide with A-L amino acids. Some other low molecular mass dipeptides were M-G (0.206 kDa, accession number OJA20730.1) found in *Rhizopogon vesiculosus*, R-G (0.231 kDa, accession number RDB21682.1) found in *Hypsizygus marmoreus,* and M-V (0.248 kDa, accession number SCN86923.1) found in *Fusarium fujikuroi*. A low molecular weight tripeptide was A-L-K (0.303 kDa, accession number KUM56815.1) and tetrapeptide was M-G-G-T (0.364 kDa, accession number PNY18451.1). The present study is the first to identify A-L, M-G, and R-G dipeptides; A-L-K tripeptides; and M-G-G-T tetrapeptides in the fungal kingdom. These small peptides can function as novel bioactive molecules that regulate several biological processes^42–44^. Small peptides can act as a hormone^45,46^, biocide^47^, and as an anti-cancer therapeutic agent^48,49^. The presence of these small peptides and their activity can have a significant impact on the physiology of an organism. Therefore, the function of these small compounds should be further explored for their potential application and commercialization.

### Isoelectric points (pI) of the fungal proteome ranges from 1.85 to 13.759

**Figure 1:**
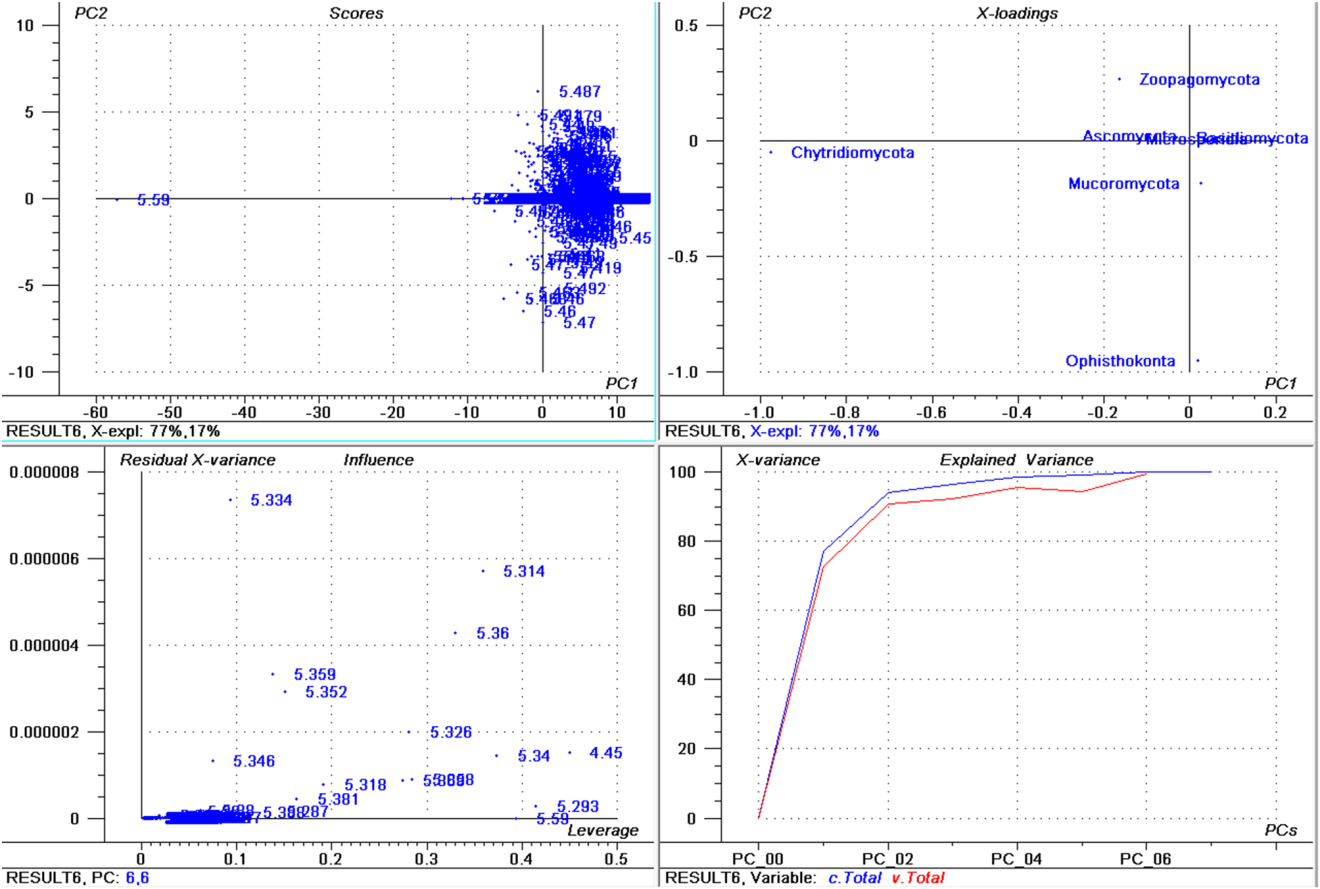
Principal component analysis (PCA) of acidic pI proteins in the fungal proteome. The figure illustrates the relationship between the acidic pI of Ascomycota, Basidiomycota, Microsporidia, Zoopagomycota, Chytridiomycota, and Opthisthokonta. The acidic pI of Chytridiomycota and Opthisthokonta cluster independently from the other phyla. Within the figure (**a**) scores: reflects the similarities in sample grouping, (**b**) loading: represents the relative position of a variable and how it relates a sample to different variables, (**c**) influence plot: represents the Q- or F-residuals vs Leverage or Hotelling T2 statistics that shows the residual statistics on the ordinate axis of sample distance to model and (**d**) variance: represent the variation in the data of different components. Total residual variance is calculated as a sum of the square of residuals for all the variables, divided by the number of degrees of freedom. The green colour indicates the calibration and the red indicates the validation.

The isoelectric point of proteins in the collective fungal proteome ranged from 1.85 in *Candida maltose* (interspersed repeat antigen (FIRA) protein, partial; accession: EMG49333.1) to 13.759 in *Ophiocordyceps sinensis* (hypothetical protein, accession: EQK98935.1). The average acidic *pI* was 5.222 while the average basic *pI* was found to be 8.489 (Supplementary File 3, Supplementary Fig. 1B,C). Among a total of 22 identified proteinogenic amino acids, the protein with the highest *pI* (EQK98935.1) was composed of only ten amino acids, Ala (63), Gly (42), Lys (21), Leu (43), Met (1), Gln (21), Arg (84), Ser (65), Val (42), and Trp (22). The Cys amino acid which is responsible for the formation of disulphide bonds in a protein was not present in the high *pI* protein. In addition, the negatively charged amino acids Asp and Glu were also not a part of the protein. Approximately 61.68% of the fungal proteins had an acidic *pI* while only 38.04% of the proteins had a basic *pI*, indicating that acidic *pI* proteins were more abundant than the basic *pI* proteins. The average of highest *pI* protein was found to be 12.446 (Supplementary Fig. 1D). When proteins with the highest *pI* in each phylum were analysed, the highest average *pI* was found in Mucoromycota (12.575) followed by Zoopagomycota (12.548), Basidiomycota (12.542), Chytridiomycota (12.497), Ascomycota (12.431), and Microsporidia (11.937). None of the species within the Microsporidia were found to encode a protein with a *pI* greater than 12.544. The average lowest *pI* among fungal phyla was found to be 2.929 (Supplementary Fig. 1E), with the lowest *pI* found in Zoopagomycota (2.441) followed by Chytridiomycota (2.64), Mucoromycota (2.749), Basidiomycota (2.897), Ascomycota (2.954), and Microsporidia (3.28). *Aspergillus ustus* was found to encode the highest percentage (76.62%) of acidic *pI* proteins, followed by *Eutypa lata* (74.58%), *Talaromyces amestolkiae* (74.16%), and *Pichia membranifaciens* (73.84%) (Supplementary File 3). *Aspergillus ustus* is a microfungus associated with human nail infections and *Eutypa lata* causes Eutypa dieback disease in grape (*Vitis vinifera*)^50^. These species belong to the phylum Ascomycota. The highest percentage of acidic *pI* proteins in the phylum Basidiomycota was found in *Malassezia vespertilionis* (68.16%) and the lowest percentage of acidic *pI* proteins was found in *Terfezia bouderi* (20.18%) followed by *Nosema ceranae* (33.49%), and *Puccinia sorghi* (33.90%) (Supplementary File 3). Among the 689 species, only 40 species (5.80%) contained basic *pI* proteins indicating that 94.19% of fungal species contain ≥ 50% acidic *pI* proteins in their proteome. A principal component analysis (PCA) of acidic *pI* proteins revealed that Ascomycota, Basidiomycota, and Microsporidia cluster together while the Zoopagomycota, Chytridiomycota, Mucoromycota, and Ophisthokonta cluster separately (Fig. 1); suggesting that the percentages of acidic *pI* protein contribute to the clustering.

**Figure 2:**
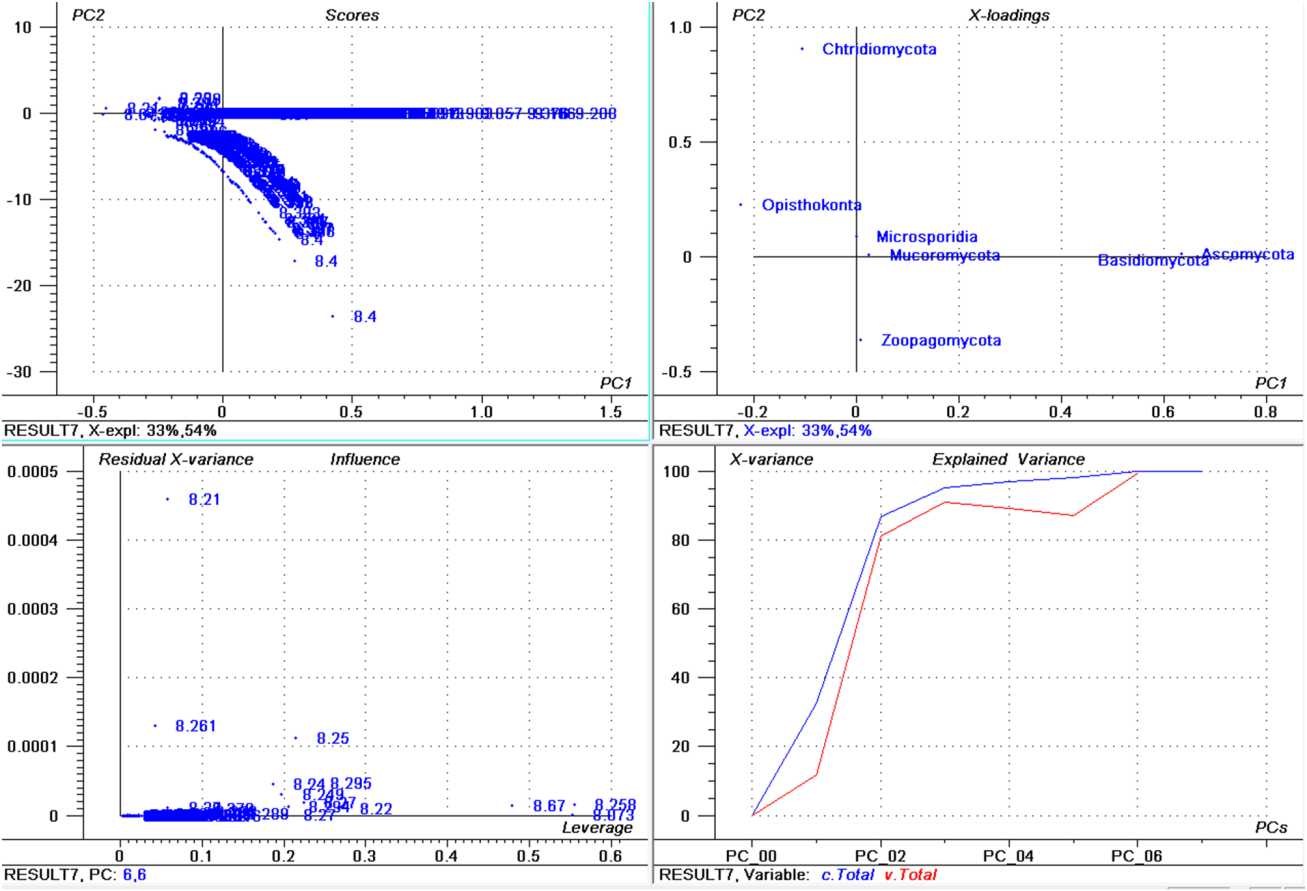
Principal component analysis (PCA) of basic pI proteins in the fungal proteome. The figure illustrates the relationship between the basic pI of Ascomycota, Basidiomycota, Microsporidia, Zoopagomycota, Chytridiomycota, and Opthisthokonta. The basic pI of Chytridiomycota, Opthisthokonta, and Zoopagomyota cluster independently from the other fungal phyla. In the figure (**a**) scores: reflects the similarities in sample grouping, (**b**) loading: represents the relative position of a variable and how it relates a sample to different variables, (c) influence plot: represents the Q- or F-residuals vs Leverage or Hotelling T2 statistics that shows the residual statistics on the ordinate axis of sample distance to model, and (**d**) variance: represent the variation in the data by different components. Total residual variance is calculated as a sum of square of residuals for all the variables, divided by the number of degrees of freedom. The green color indicates the calibration and the red indicates the validation. The collective fungal proteome was found to contain an average of 17.449 (0.172%) neutral *pI* proteins (Fig. 1F). The *pI* of the entire plant kingdom has been reported to range from 1.99 to 13.96^42^. The lowest *pI* found for fungal proteins was less than the lowest plant protein *pI*. In contrast, the highest *pI* found for a fungal protein was lower than the highest *pI* found for a plant protein.

**Figure 3:**
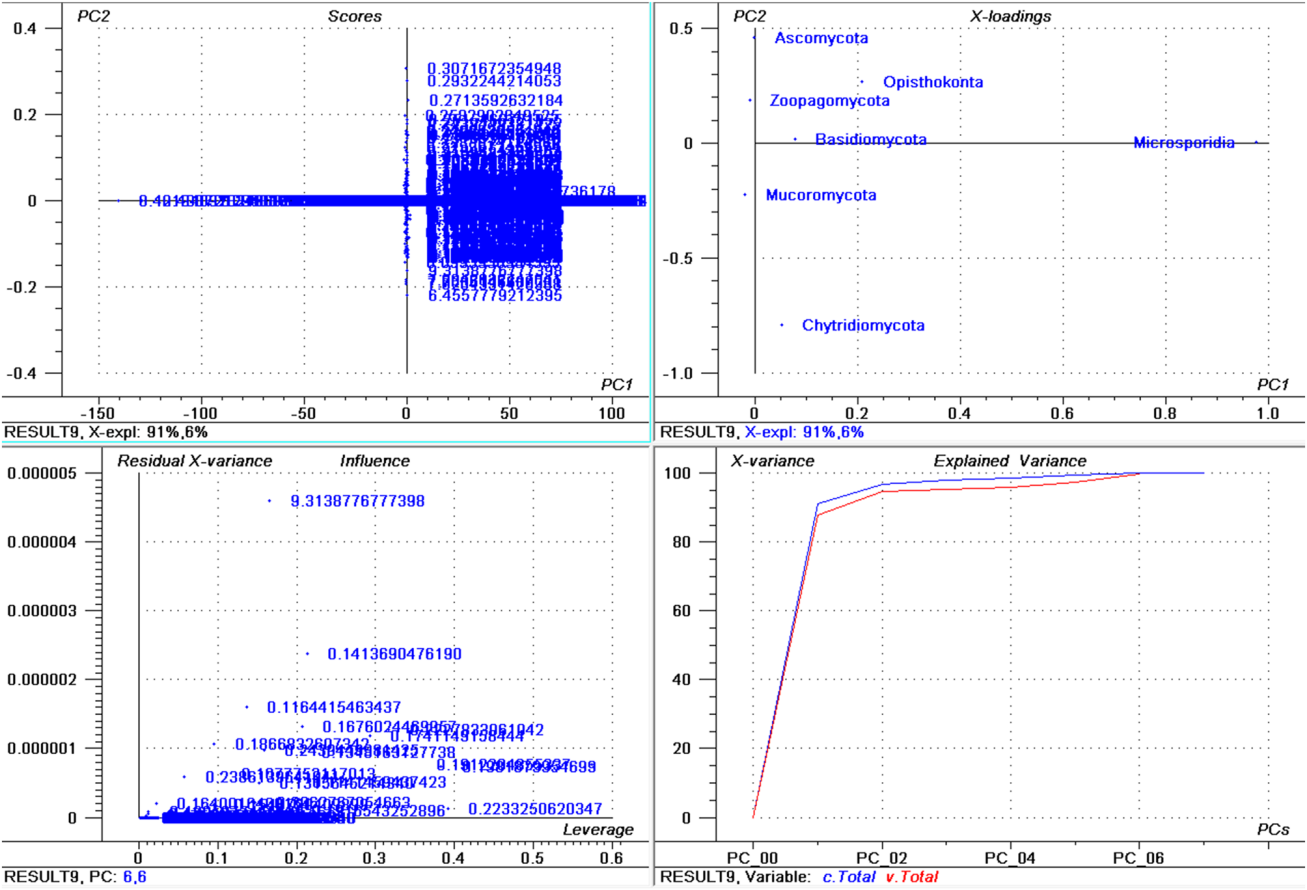
Principal component analysis (PCA) of percentage (%) of occurrence of neutral pI proteins in the fungal proteome. The figure illustrates the relationship between the percentage of neutral pI proteins in the Ascomycota, Basidiomycota, Microsporidia, Zoopagomycota, Chytridiomycota, and Opthisthokonta.

The highest percentage of basic *pI* proteins was found in *Nosema ceranae* (66.15%) followed by *Puccinia sorghi* (65.89%), and *Tubulinosema ratisbonensis* (63.55%) (Supplementary File 1). *Nosema ceranae* is the causal agent of nosemosis (a disease in honey bees) and its spores are resistant to high temperature and dehydration. This fungus also produces an insecticide, similar to fipronil/nicotinoids, which causes male sterility in honey bees^51,52^. The lowest percentage of basic *pI* proteins was found in *Aspergillus ustus* (23.22%), followed by *Eutypa lata* (25.26%), and *Fonsecaea erecta* (25.59%) (Supplementary File 3). The highest percentage (≥ 60%) of basic *pI* proteins was found in species within the Microsporidia and Basidiomycota group. In contrast, none of the species in the Ascomycota was found to encode ≥ 60% basic *pI* proteins in it. The PCA of basic *pI* proteins revealed that Microsporidia and Mucoromycota cluster together while Ascomycota and Basidiomycota cluster together (Fig. 2). However, Chytridiomycota, Ophisthokonta, and Zoopagomycota cluster independently (Fig. 2). This suggests that the basic *pI* proteins in the Microsporidia with Mucoromycota and Ascomycota with Basidiomycota share a common pattern of basic *pI*. Notably, the PCA of the percentage of neutral *pI* proteins did not exhibit any distinct clustering of phyla (Fig. 3). All of the groups appeared to locate independently from each other, suggesting a great diversity in the percentage of neutral *pI* proteins in the fungal proteome.

### The Mol. Weight and pI of fungal proteins exhibit a bimodal distribution

**Figure 4:**
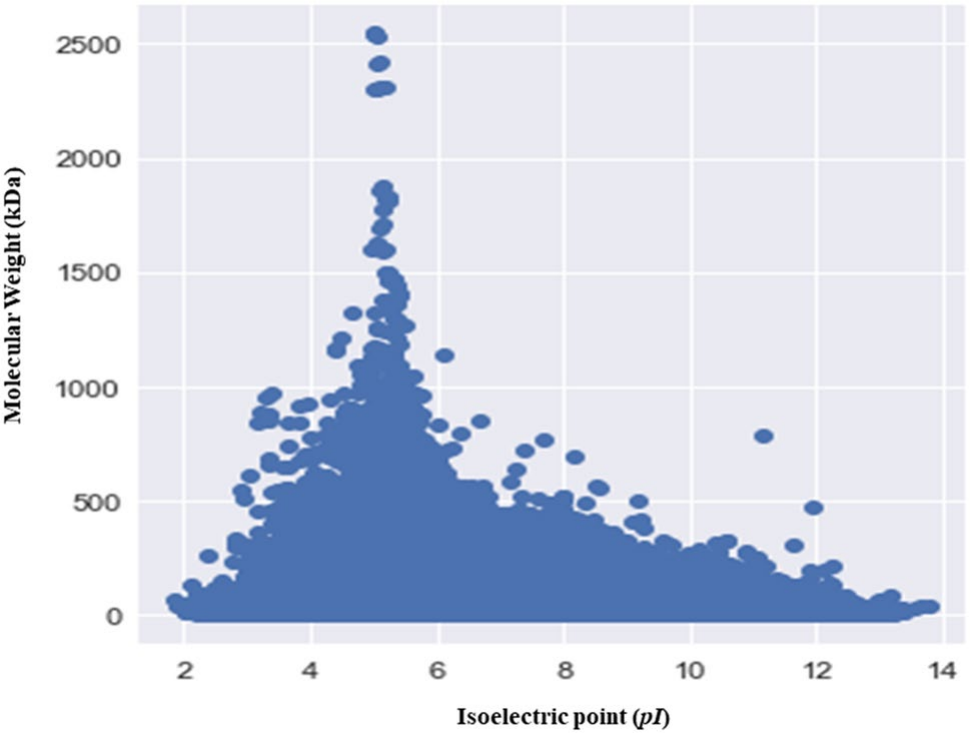
Virtual 2D map of the fungal proteome. The virtual map reveals the bimodal distribution of molecular mass (kDa) and isoelectric point (pI) in the collective fungal proteome. The X-axis represents the isoelectric point and Y-axis represents the molecular mass of the fungal proteins.

The *pI* of the analysed fungal proteins, ranging from 1.85 to 13.759 and exhibited a bimodal distribution (Fig. 4). The bimodal distribution of the *pI* and molecular mass of the fungal proteome provides a virtual 2-D proteome map of the fungi (Fig. 4). Mohanta et al., (2019) previously reported a trimodal distribution of plant proteomes^42^. Schwartz et al., also reported a trimodal distribution of the *pI* of eukaryotic proteins^37^. The *pH* of cytoplasm is close to neutral while the majority of fungal proteins fall in the acidic *pI* range (Fig. 4). Approximately 94.21% of the fungal species encode proteins whose proteome contain more than 50% of acidic *pI* proteins. The *pI* peak of acidic proteins was more prominent compared to basic and neutral proteins (Fig. 4). Kiraga et al., (2007) reported that acidic and basic *pI* properties of the proteins are the basis of bi- and trimodal distribution^53^. They also reported that taxonomy, ecological niche, and sub-cellular localization play an important role in determining the acidity and basicity of a protein^53^. Therefore, we analysed the normal probability distribution of the percentage of all of the acidic *pI* proteins from all fungal phyla (Ascomycota, Basidiomycota, Chytridiomycota, Microsporidia, Mucoromycota, and Zoopagomycota). Results indicated that the correlation coefficient (0.9635) tended slightly towards linearity, irrespective of taxonomical hierarchy (Supplementary Fig. 3A). Similarly, the normal probability distribution of the percentage of basic *pI* proteins in all taxonomical groups of fungi was calculated. Results indicated that the correlation coefficient was 0.974 (Supplementary Fig. 3B). Basic *pI* proteins were correlated in a greater extent with their number and percentage in the collective fungal proteome compared to the acidic *pI* proteins. No correlation was found between acidic or basic *pI* proteins and their taxonomy. Knight et al.^54^ also reported a negative correlation between the *pI* and taxonomy of an organism as well as a negative correlation between the *pI* of proteins and the phylogeny of an organism^54^. Only 0.172% of the proteins in fungi were found in the neutral *pI* range (Supplementary File 3). *Tilletia controversa* had the highest percentage (2.231%) of neutral *pI* proteins and *Anncaliia algerae* had the lowest percentage (0.027%) of neutral proteins (Supplementary File 3).

The provided molecular mass and *pI* of the fungal proteomes are based on the annotated protein sequences. During the event of protein translation, the protein sequences undergo post-translational modification including methylation, phosphorylation, acetylation, hydroxylation, amidation, ubiquitylation, sulfation, glycosylation, and other^55,56^. These post-translational modification event changes the charges of the protein molecule^57^. Therefore, the predicted *pI* of the protein may be little different from the exact *pI*. Kumar et al., (2004) reported that the extent of shift in *pI* of a protein upon phosphorylation is dependent on its size and native *pI*^58^. Therefore, the native *pI* of a protein plays critical role as well. The shift of *pI* of a protein in a particular posttranslational event also vary greatly^58^. A protein with lower molecular weight tend to show *pI*-shift more frequently than the higher molecular weight proteins^58^. Addition of at least phosphorylation to a protein with a molecular mass of 110 kDa result in a *pI*-shift of less than 0.2 units and an addition of the same amount of phosphorylation with a molecular weight of 11.8 kDa result in a *pI-*shift of approximately 1 unit^58^. Protein with basic *pI* show maximum *pI-*shift upon phosphorylation while acidic *pI* protein shows a relatively lower *pI*-shift^58^. The fungal proteome is predominated by acidic *pI* proteins compared to the basic *pI* proteins. Therefore, the *pI*-shift of fungal acidic *pI* proteome will be relatively lower. However, phosphorylation and dephosphorylation of a protein is a continuous process and none of a particular protein ever stay in either at phosphorylation or dephosphorylation state. Therefore, the *pI*-shift due to change in phosphorylation also revert back to the original state upon dephosphorylation. Cathepsin B protein upon single phosphorylation shifts its *pI* from 5.23 to 5.15 (experimental)^59^ and heat induced nuclear accumulation protein HSC70 upon single phosphorylation shifts it *pI* from 5.48 to 5.43 (experimental)^60^.

### Leu is a high-abundant and Cys is a low-abundant amino acid in the Fungal Kingdom

The amino acid composition of proteins in the fungal kingdom was analysed to determine which amino acids were more abundant and which were lower in abundance (Supplementary File 1). It was estimated that fungi encoded 4.675520004 million amino acids per proteome. The proteome of *Fibula rhizoctonia* encoded highest number of amino acids (10.940068 million) followed by *Sphaerobolus stellatus* (10.818606 million), *Rhizophagus clarus* (10.524515 million), and *Diversispora versiformis* (10.170037 million) (Supplementary File 2). The analysis revealed that Leu (9.115%) was the most abundant amino acid in the collective fungal proteome while Cys (1.267%) was the least abundant (Table 1). The high abundance of Leu was followed by Ser (8.465%), Ala (8.066%) and Gly (6.41%); while the low abundance of Cys (1.267%) was followed by Trp (1.322%) (Table 1). As Leu, Ala, and Gly are non-polar amino acids, it seems the fungal proteome favours the synthesis of non-polar amino acids than other types. It would be very interesting to determine the reason behind the differential abundance of amino acids in the fungal proteome. When the half-life of individual amino acids was compared, Leu was found to have the shortest half-life (namely, 3 min), while the half-life of Cys was more than 20 h^61–64^. Since Leu has very short half-life period, it needs to encode a greater number of Leu amino acid in the cell to maintain protein synthesis machinery. Cys has a very long half-life and thus the degradation of Cys in the cell is relatively slower than Leu. Therefore, it seems that fungal cells synthesize a lower number of Cys amino acids. Cys amino acid used to produce glutathione anti-oxidant as well as amino acid taurine. Cys also sometimes converted to glucose for the source of energy^65^. A cross-talk between Cys and glutathione is critical for the regulation of amino acid signaling pathway^66^. The role of Cys as the precursor molecule in plant is also highly important other than its role in the protein^67^. Its role in plant stress response is highly important for its redox activity^67^. Accumulation of Pro amino acid occurs due to osmotic stress in plants and it act as a plant abiotic stress marker^68^. However, accumulation of other amino acids like Asp, Glu, Ser, Gly, and Gln also occur due to biotic and abiotic stress responses^69^.

**Table 1:** Amino acid composition of the fungal proteomes.

Amino acids	Composition (%)
Leu	9.115056391
Ser	8.465427891
Ala	8.066886639
Gly	6.416593956
Glu	6.212047906
Val	6.080098604
Thr	5.885447967
Arg	5.79174785
Pro	5.70190633
Asp	5.677441609
Lys	5.309656746
Ile	5.291616955
Asn	4.128360538
Gln	3.960659614
Phe	3.856287715
Tyr	2.889458344
His	2.388615762
Met	2.156579159
Trp	1.32229263
Cys	1.267849975
Xaa	0.015967419

**Figure 5:**
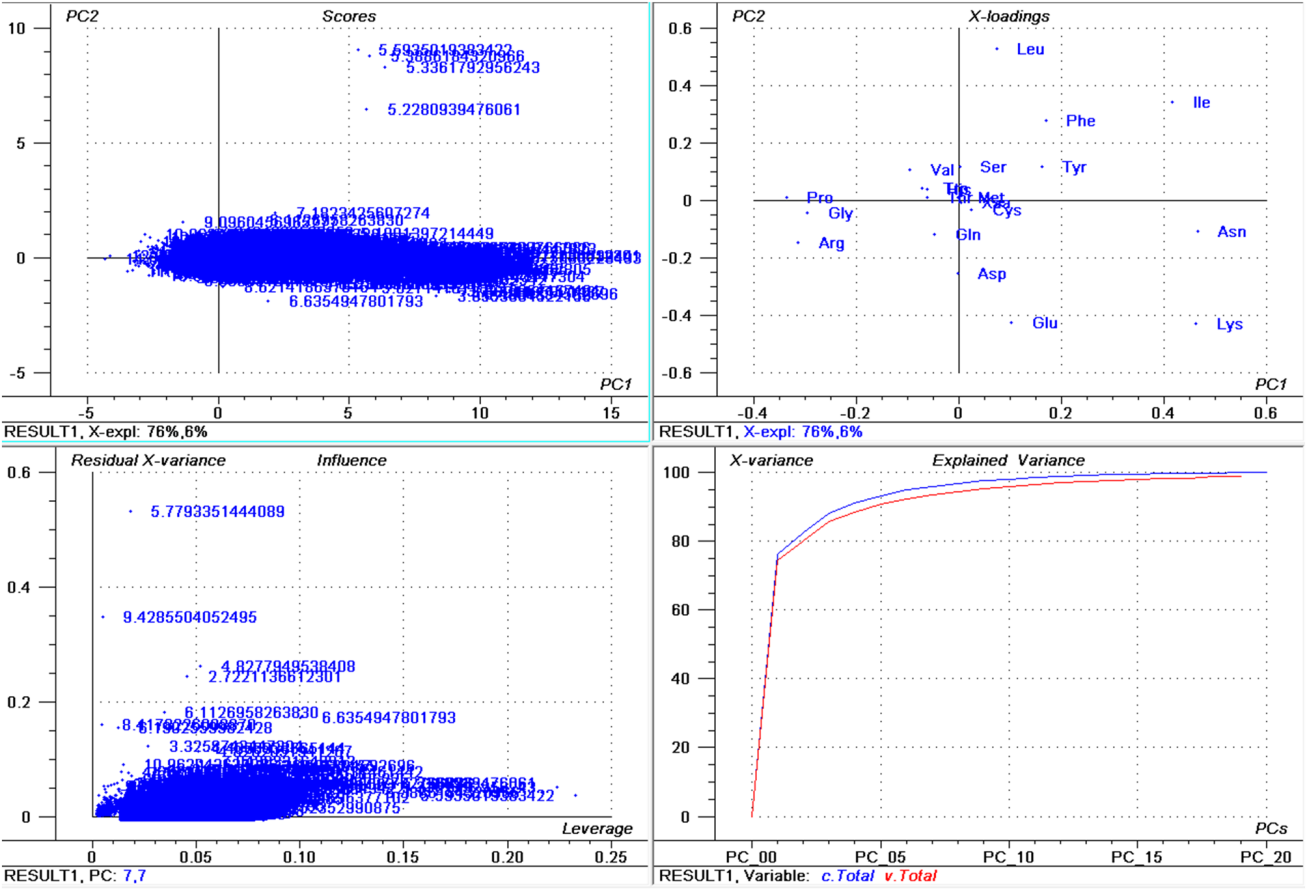
PCA analysis of amino acid composition in fungal proteomes. Analysis revealed that Pro, Gly, and Arg; Val, Ser, Met, Cys, Gln, Trp, Thr, His, and Ala cluster together while Leu, Ile, Phe, Tyr, Asn, Asp, Glu, and Lys group independently from the other amino acids.

PCA analysis of the amino acid composition of fungal proteins revealed that Cys, Met, Xaa (unknown amino acids), Thr, Trp, Val, and Ser clustered together while Leu, Ile, Phe, Asn, Lys, Glu, and Ala locate separately (Fig. 5). Further analysis indicated that the highest percentage of Ala (14.00%) and Arg (7.76%) was found in *Tilletiopsis washingtonensis* and the highest percentage of Cys (2.40%), Met (3.78%), and Val (7.29%) was found in *Ordospora colligate* (Table 2). The lowest percentage of Phe (2.842%), Ile (3.039%), Lys (3.359%), and Asn (2.139%) was found in *Tilletiopsis washingtonensis,* while the lowest percentage of Cys (0.533%), Asp (3.023%), Gln (1.956%), and Arg (2.383%) was found in *Trichoderma asperellum* (Table 2).

**Table 2:** Highest and lowest composition of amino acids in the fungal kingdom.

Amino acids	Highest percentage (%)	Name of the species with highest abundance	Lowest percentage (%)	Name of the species with lowest abundance	Average percentage (%)	Standard deviation	variance
Ala	14.009	*Tilletiopsis washingtonensis*	2.315	*Nosema apis*	8.066	1.678	0.593
Cys	2.407	*Ordospora colligata*	0.533	*Trichoderma asperellum*	1.267	0.193	0.039
Asp	6.428	*Pichia membranifaciens*	3.023	*Trichoderma asperellum*	5.677	0.302	0.016
Glu	9.651	*Amphiamblys sp*	2.836	*Penicillium roqueforti*	6.212	0.519	0.203
Phe	7.845	*Penicillium nordicum*	2.84	*Tilletiopsis washingtonensis*	3.856	0.535	0.396
Gly	8.035	*Jaminaea rosea*	3.00	*Edhazardia aedis*	6.416	0.87	0.073
His	3.300	*Malassezia restricta*	1.471	*Hepatospora eriocheir*	2.388	0.233	0.025
Ile	11.170	*Trichoderma asperellum*	3.039	*Tilletiopsis washingtonensis*	5.291	1.174	1.779
Lys	11.063	*Tubulinosema ratisbonensis*	3.359	*Tilletiopsis washingtonensis*	5.309	1.293	1.920
Leu	14.101	*Penicillium roqueforti*	7.753	*Piromyces sp.*	9.115	0.523	0.351
Met	3.787	*Ordospora colligata*	1.462	*Ascoidea rubescens*	2.156	0.189	0.032
Asn	10.944	*Anaeromyces robustus*	2.139	*Tilletiopsis washingtonensis*	4.128	1.29	2.366
Pro	7.272	*Cutaneotrichosporon oleaginosum*	2.00	*Enterocytozoon hepatopenaei*	5.701	0.954	0.071
Gln	5.278	*Absidia repens*	1.956	*Trichoderma asperellum*	3.960	0.37	0.050
Arg	7.766	*Tilletiopsis washingtonensis*	2.383	*Trichoderma asperellum*	5.791	0.893	0.078
Ser	11.474	*Smittium mucronatum*	6.322	*Enterocytozoon bieneusi*	8.465	0.61	0.233
Thr	6.776	*Absidia glauca*	4.057	*Encephalitozoon cuniculi*	5.885	0.332	0.014
Val	7.295	*Ordospora colligata*	4.315	*Ascoidea rubescens*	6.080	0.407	0.035
Trp	1.647	*Nectria haematococca*	0.431	*Edhazardia aedis*	1.322	0.227	0.001
Tyr	5.081	*Spraguea lophii*	0.053	*Candida orthopsilosis*	2.889	0.495	0.261
Xaa	1.719	*Rhizoctonia solani*	0.000		0.015	0.115	

The lowest percentage of Gly (3.00) and Trp (0.431) was found in *Edhazardia aedis,* while the lowest percentage of Met (1.462%) and Val (4.315%) was found in *Ascoidea rubescens* (Table 2). Amino acid abundance was also examined by grouping species into different phyla. Results indicated that amino acid abundance tended to be phyla-specific (Table 3).

**Table 3:** Distribution of average amino acid composition (%) in different groups of the fungal kingdom.

Amino acids	Ascomycota	Basidiomycota	Microsporidia	Chytridiomycota	Mucoromycota	Zoopagomycota
Ala	8.1	9.06	4.37	6.82	6.63	6.83
Cys	1.23	1.22	1.89	1.44	1.41	1.33
Asp	5.72	5.58	5.39	5.59	5.79	5.52
Glu	6.22	5.92	7.54	6.24	6.22	6.08
Phe	3.86	3.54	5.18	3.87	3.98	4.05
Gly	6.54	6.66	4.62	5.56	5.18	5.72
His	2.35	2.56	1.93	2.21	2.64	2.22
Ile	5.24	4.62	8.27	6.32	6.06	6.13
Lys	5.26	4.5	8.85	6.44	6.19	6.24
Leu	9.07	9.19	9.53	8.78	9.12	9.18
Met	2.15	2.07	2.46	2.16	2.39	2.18
Asn	4.11	3.34	6.43	6.08	5	5.51
Pro	5.68	6.46	3.05	5	4.97	4.86
Gln	4.02	3.87	2.88	3.76	4.62	3.93
Arg	5.76	6.34	4.56	4.75	5.06	4.99
Ser	8.37	8.88	7.45	8.58	8.28	9.2
Thr	5.91	5.95	4.97	5.97	6.06	5.75
Val	6.08	6.19	5.8	5.96	5.75	5.86
Trp	1.35	1.39	0.66	1.08	1.19	1.04
Tyr	2.88	2.54	4.01	3.3	3.36	3.28

### Fungi do not encode selenocysteine (Sec) and pyrrolysine (Pyl) amino acids

Selenocysteine (Sec) and Pyrrolysine (Pyl) are considered proteinogenic amino acids. These amino acids were found to be absent in any of the 7.15 million fungal protein sequences examined so far. A selenoproteomic study in yeast also did not find the presence of Sec^70^. The presence of some unknown amino acids (Xaa) was also found in our analysis (Supplementary File 1). On average, 0.015% (statistically insignificant) of fungal proteins contain a Xaa amino acid. The highest percentage (1.719%) of Xaa amino acids was found in *Rhizoctonia solani* followed, by *Brettanomyces bruxellensis* (1.64%), and *Trachipleistophora hominis* (1.19%) (Supplementary File 1). Among the 689 examined species, 317 contained Xaa amino acids. In addition to Xaa amino acids, a few species also encoded ambiguous amino acids B, J, and Z amino acids (Supplementary File 1). The highest number of B (1415), J (363), and Z (405) amino acids was found in *Brettanomyces bruxellensis, Hanseniaspora opuntiae*, and *Hanseniaspora uvarum,* respectively (Supplementary File 1). The ambiguous amino acid B stands for either Asn or Asp and translates into Asn; J stands for Leu or Ile and translate into Leu; and Z stands for Gln or Glu and translates into Gln. Previous studies by Mariotti et al., (2019) and Jiang et al., (2012) also reported the absence of Sec in fungal proteins^71,72^. Mariotti et al., (2019), however, have reported that fungi do contain Sec amino acids^73^. They reported the presence of Sec amino acids encoding machinery in nine species *Bifiguratus adelaide, Gonapodya prolifera, Capniomyces stellatus, Zancudomyces culisetae, Smittium angustum, and Furculomyces boomerangus*^73^. This stands in contrast to the findings of the present study for its translated product. To further validate the findings of the present study, we re-analysed the proteome sequences of all the nine species mentioned by Mariotti et al., (2019) but did not find the presence of any Sec (U) amino acids (Supplementary Fig. 4). Mariotti et al., (2019) utilized a BLASTN approach in their study, which might be the cause of the noted contradiction^73^. When we searched for Sec-encoding protein sequences in the 7.15 million fungal proteome sequences from 689 species, we found that 134 sequences in 112 fungal species had amino acid sequences with an annotation name “selenocysteine” (Supplementary File 4). When we downloaded and searched 134 protein sequences for the presence of Sec amino acid, none of the sequences were found to contain Sec amino acid in it (Supplementary Fig. 4). A protein should not be referred to as a selenoprotein if it does not contain any Sec amino acid in it. Therefore, the protein sequences annotated as “selenocysteine” cannot be considered as a selenoprotein. These contradictions may be the result of an annotation error. Similarly, we also did not find the presence of the proteinogenic amino acid, Pyl encoded by a UAG codon, in any of the fungal proteins analysed so far^74^. Development of high throughput annotation pipeline can enable us to find the presence of Sec and Pyl amino acids in the fungal proteome in more details.

### Fungi encode fewer proteins than plants and animals

**Figure 6:**
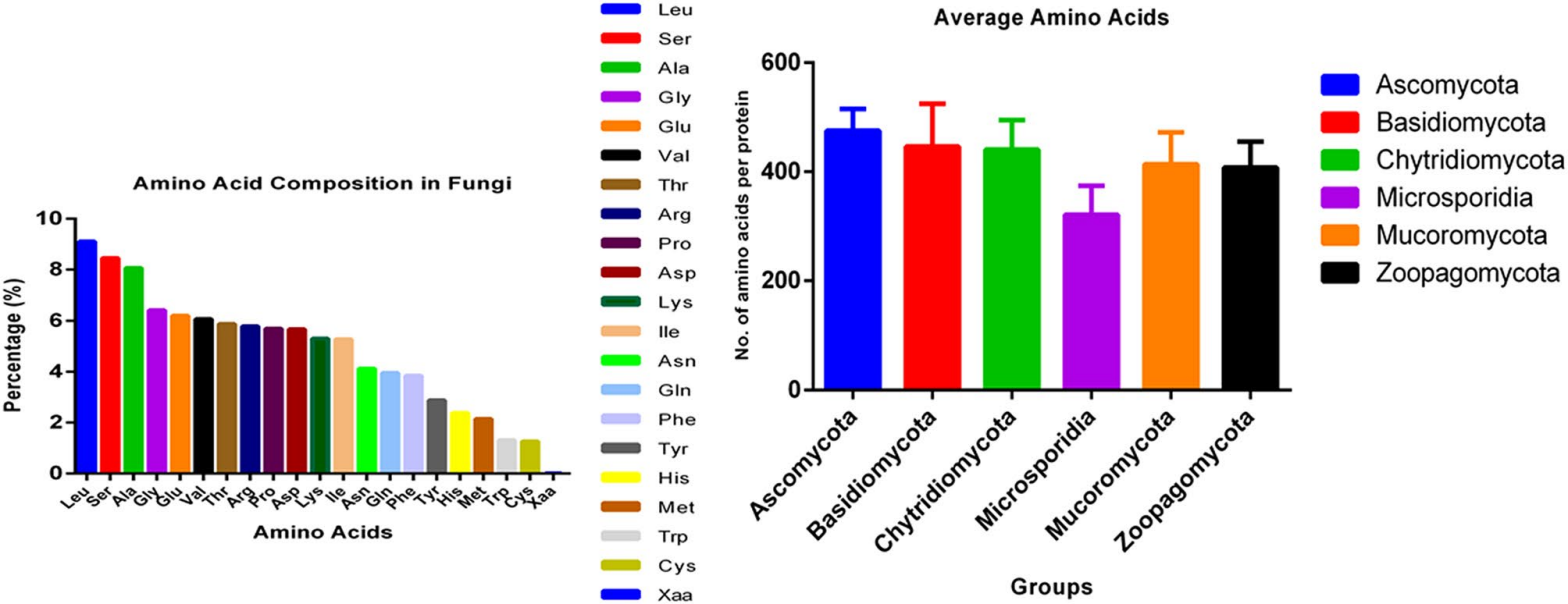
Average number of amino acids per protein in the fungi. The protein sequences of species within the Ascomycota possess a higher number of amino acids than was found in the species of other phyla. Protein sequences of species within the Microsporidia possess a lower number of amino acids.

Our analysis revealed that fungal proteomes contain an average of 10,345.83 protein sequences per species. Fungal species were previously reported to contain an average of 9113 protein sequences per species^75^. The previous study, however, was limited to only 143 species which may be the reason why a lower number of protein sequences per species was reported. Plants encode an average of 40,469.47 proteins per species and animals 25,189 per species^42^. The average of 40,469.83 protein sequences in plants is 391.166% higher than fungi and the average of 25,189 protein sequences per species in animals is 243.47% higher than fungi. This indicates that both plants and animals encode a higher number of protein sequences in their proteome than fungi. No fungal species was found to encode ≥ 40,000 protein sequences in their proteome. The average number of amino acids per fungal protein sequence was calculated to be 459.319. The average number of amino acids per protein sequence in the Ascomycota, Basidiomycota, Chytridiomycota, Microsporidia, Mucoromycota, and Zoopagomycota was 475.406, 446.155, 440.876, 321.063, 413.661, and 407.517, respectively. The average number of amino acids per protein in the Ascomycota species was the highest while the lowest average number was found in Microsporidia (Fig. 6). The average length of plant proteins was reported to be 424.34 amino acids per protein while the average length of all eukaryotic proteins was reported to be 472 amino acids per protein^42,75,76^. The presence of 459.319 amino acids per protein in fungi falls between the average protein size reported for plants and for all eukaryotic organisms. Longer proteins contain a greater number of conserved domains and display more biological functions. The presence of a lower average number of protein sequences in fungi compared to plants and animals may be the reason that fungi contain a higher number of amino acids per protein. Although the average size of proteins in plants is smaller than in fungi, the protein size is higher in unicellular plant species, such as *Chlamydomonas eustigma* (576.56), *Volvox carteri* (568.22), *Klebsmordium nitens* (538.73), *Durio zibethinus* (504.36), and *Bathycoccus prasinus* (521.05)^42^. This indicates that the average protein size in unicellular organisms is larger than in multicellular organisms (plant, fungi, and animals) and suggests that protein evolution is associated with a reduction in average protein size. Although it is difficult to delineate the exact reasons for the reduction in protein size, evolutionary factors contributing to the reduction include the deletion of exons and/or the fusion of multiple protein domains, both of which may have played a role in determining the size of fungal proteins. In addition, we also did not find any correlation between protein size and *pI*. Larger proteins are not proportionately associated with a higher *pI*. Previous studies have reported that the reduction in protein size in the plant genome was partially due to endosymbiosis^42,75,77,78^. Based on this hypothesis, it seems plausible that the heterotrophic (parasitic and saprophytic) and symbiotic life of fungi may have played an important role in the reduction in protein size that has occurred in the fungi, relative to unicellular plants. The physical structure of a protein is greatly influenced by its primary structure, i.e. the number, composition, and order of the amino acids it contains. Although the chemical environment surrounding a protein plays an important role, the primary sequence of a protein is essential in determining its function. Protein size is an important biochemical parameter as longer proteins can accommodate a greater number of functional protein domains and can therefore display more biological functionality. Shorter proteins/polypeptides possess a limited number of functionalities, while larger proteins, with more functional domains, can serve multiple regulatory functions.

## Conclusions and future perspectives

A virtual 2-D map of fungal proteomes was constructed and used to evaluate the range of *pI* and molecular masses present in the proteome of the fungal kingdom. The analysis of the fungal proteome indicated that it does not encode pyrrolysine and selenocysteine amino acids. The average molecular mass of fungal proteins was greater than the average molecular mass of plant proteins, suggesting fungal proteomes are evolutionarily older than plant proteomes. The fungal proteome was dominated by acidic *pI* proteins, however, the higher percentage of the basic *pI* proteins in *Nosema ceranae* may be important and warrants further evaluation to understand its molecular aspects of high *pI* and possible potential application. Basic *pI* proteins provide extra positive charges and can serve as an excellent system to study how cells function and operate using a greater number of basic *pI* proteins. The amino acid composition of the fungal protein will allow us to understand the synonymous codon usage in the protein coding gene and its subsequent evolution. The study can act as a valuable tool for peptide mass finger printing (PMF) and allow to understand the theoretical mass of the peptides. Using this principle, we can understand the molecular mass and *pI* of sub-proteome and secretome as well. Filamentous fungi secrete a vast number of secreted proteins to carry out their saprophytic lifestyle. Molecular mass and *pI* of secreted proteins can be valuable to identify the secreted proteins in other organisms. The amino acid composition of fungal proteomes was deciphered using straightforward protein sequences which is useful for finding the homology proteins of known characteristics from an unknown species. However, the sequence similarity model sometimes might fail to work when the query protein did not have significant sequence similarity with the known protein. In this case, the molecular weight and or *pI* can be very helpful to understand possible function of the protein including its sub-cellular localization.

At the present, we are in the post-genomic era and we have avalanche of protein sequences. We must able to use the newly found novel protein sequences for basic research and biotechnological applications including drug development in large-scale manner. This protein universe of cells can be merged with the systems biology so that it can be incorporated into the gene regulatory network of integrated protein switches. A successful story can be achieved on modular interactions between protein domains and peptide motifs through the integrated approach of molecular weight and pI of proteins. It will be interesting to understand the engineering of single amino acid residue rather than engineering proteins in the cell to understand the role of hyper or hypo amino acid abundance of particular amino acid and its synonymous codon usage and tRNA supply. This will reveal a great deal of evolutionary consequences of synonymous codon usage, tRNA supply, and abundance of particular amino acid.

## Material and methods

### Sequence retrieval and calculation of molecular weight and *pI*

The annotated proteome files of the 689 species of fungi deposited in the National Center for Biotechnology Information (NCBI) were downloaded and used for the analyses. The number of amino acids (amino acid composition) in each of the proteome files was determined using Linux-based commands. Molecular masses and isoelectric points of all the proteins in the annotated files were individually analyzed using the IPC in the python module of “protein isoelectric point calculator”^79^. The generated files for the isoelectric point and molecular mass of the individual species was subsequently converted to an excel file format. All of the quantitative analyses (highest and lowest molecular mass and isoelectric point, composition and abundance of amino acids) were conducted in Microsoft Excel 2016. The virtual 2-D proteome map was constructed using the python-based platform by considering the isoelectric point and molecular weight of 7.15 million protein sequences (x-axis: count 7.209672e + 06 7.209672e + 06, mean: 6.672264e + 00 5.013158e + 01, std: 1.693270e + 00 4.082312e + 01, min: 1.850000e + 00 2.940000e − 03, 25%: 5.347000e + 00 2.371829e + 01, 50%: 6.160000e + 00 4.072890e + 01, 75%: 7.995000e + 00 6.307570e + 01, and max:1.375900e + 01 2.546167e + 03).

### Statistical analysis

The principal component analyses (PCA) of the plant proteome were carried out using the statistical software, Unscrambler version 9.7, using the required data from the Excel files. To determine different details on the acidic, basic, and neutral *pI* of proteins, the data were grouped into Ascomycota, Basidiomycota, Blastocladiomycota, Chytridiomycota, Glomeromycota, Microsporidia, Mucoromycota, Neocallimastigomycota, Ophisthokonta, and Zoopagomycota. The regression analysis, probability plot for the amino acid number, normal probability distribution for acidic and basic *pI* proteins (%), and Pearson correlation for amino acids were conducted using Past3 software version 1.0.0. Box and whisker plots were constructed using Microsoft Excel 2016.

## Supplementary information

Supplementary information 1.

Supplementary information 2.

Supplementary information 3.

Supplementary information 4.

Supplementary information 5.
