# Supplementary material for: Virtual 2-D map of the fungal proteome
Source: Sci Rep. 2021 Mar 23;11:6676. doi: 10.1038/s41598-021-86201-6 (PMC7988114; doi:10.1038/s41598-021-86201-6)

Virtual 2-D Map of The Fungal Proteome

Tapan Kumar Mohanta[1¥*], Awdhesh Kumar Mishra[2¥], Adil Khan[1], Abeer Hashem[3,4], Elsayed Fathi Abd_Allah[5], Ahmed Al-Harrasi[1*]

[1]Natural and Medical Sciences Research Center, University of Nizwa, 616, Oman.

[2]Dept. of Biotechnology, Yeungnam University, Gyeongsan, Gyeongsangbuk-do, 38541, Republic of Korea

[3]Botany and Microbiology Department, College of Science, King Saud University, P.O. Box. 2460, Riyadh 11451, Saudi Arabia (https://orcid.org/0000-0001-6541-347X).

[4]Mycology and Plant Disease Survey Department, Plant Pathology Research Institute, ARC, Giza 12511, Egypt.

[5]Plant Production Department, College of Food and Agricultural Sciences, King Saud University, P.O. Box. 2460, Riyadh 11451, Saudi Arabia (https://orcid.org/0000-0002-8509-8953).

To whom correspondence should be addressed: Tapan Kumar Mohanta, E-mail: nostoc.tapan@gmail.com, tapan.mohanta@unizwa.edu.om; Ahmed Al-Harrasi, E-mail: aharrasi@unizwa.edu.om

Phone: +968-79405046

¥ indicates contributed equally

Supplementary Figure 1

Box and Whisker plot of (A) average number of protein sequences (10,336.43) per proteome, (B) average number of acidic pI proteins (5.522), (C) average number of basic pI proteins (8.489), (D) average number of the highest pI proteins (12.446), and (E) average number of the lowest pI proteins (2.929), (F) average number of neutral pI proteins (17.474).

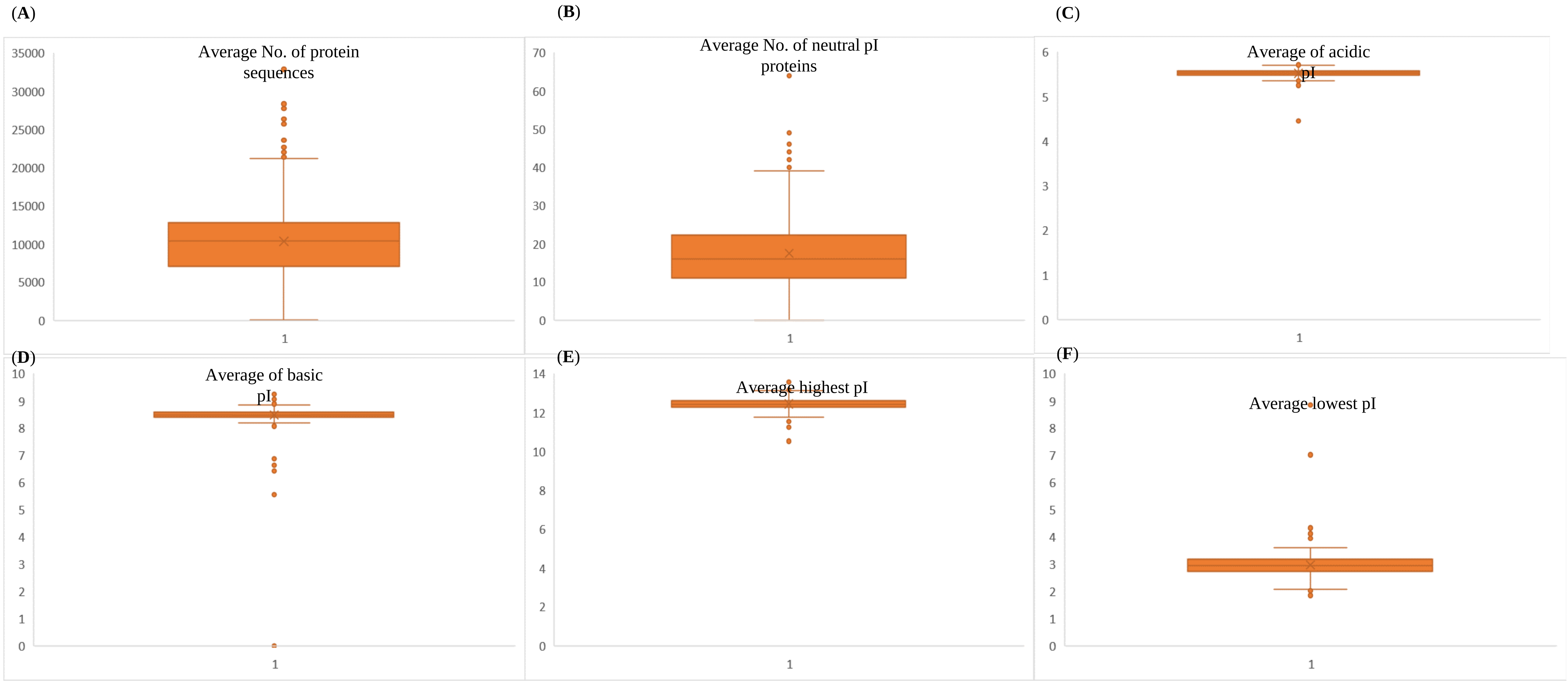

Supplementary Figure 2

(A) Correlation of genome size and number of amino acids per proteome. slope *a*: 0.1178, intercept *b*: 1.2774; 95% bootstrap confidence interval *N*=1999: slope a: 0.10084, intercept *b*=1.184, correlation: *r*=0.650, r^2=0.422, *t*=22.424, *p*(uncorr.)=5.774, permutation *p*: 0.0001). (B) Correlation coefficient of various amino acids of the fungal proteome.

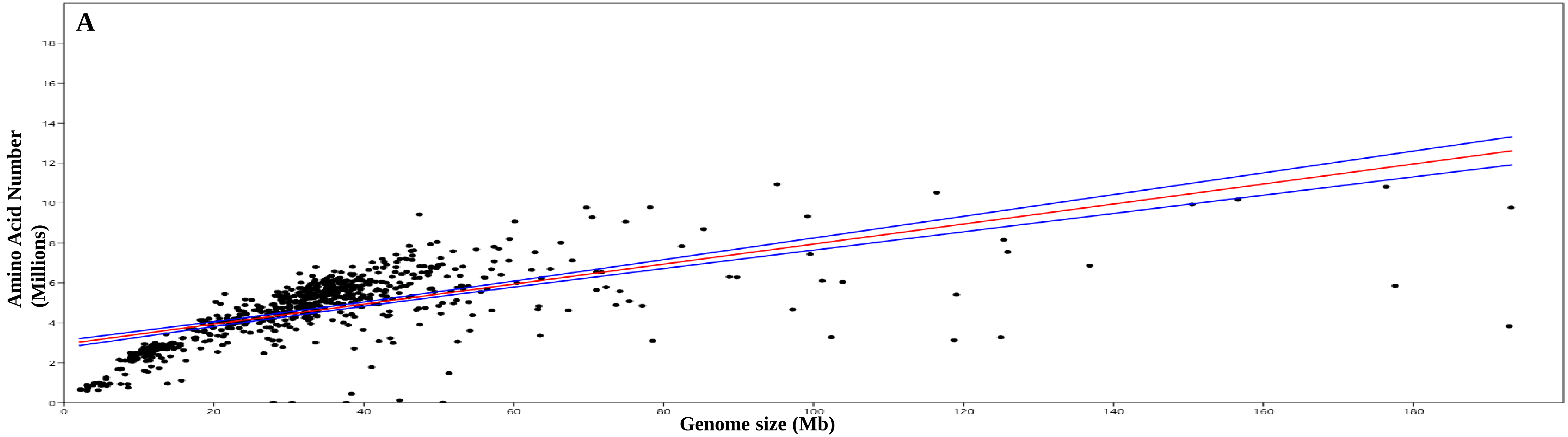

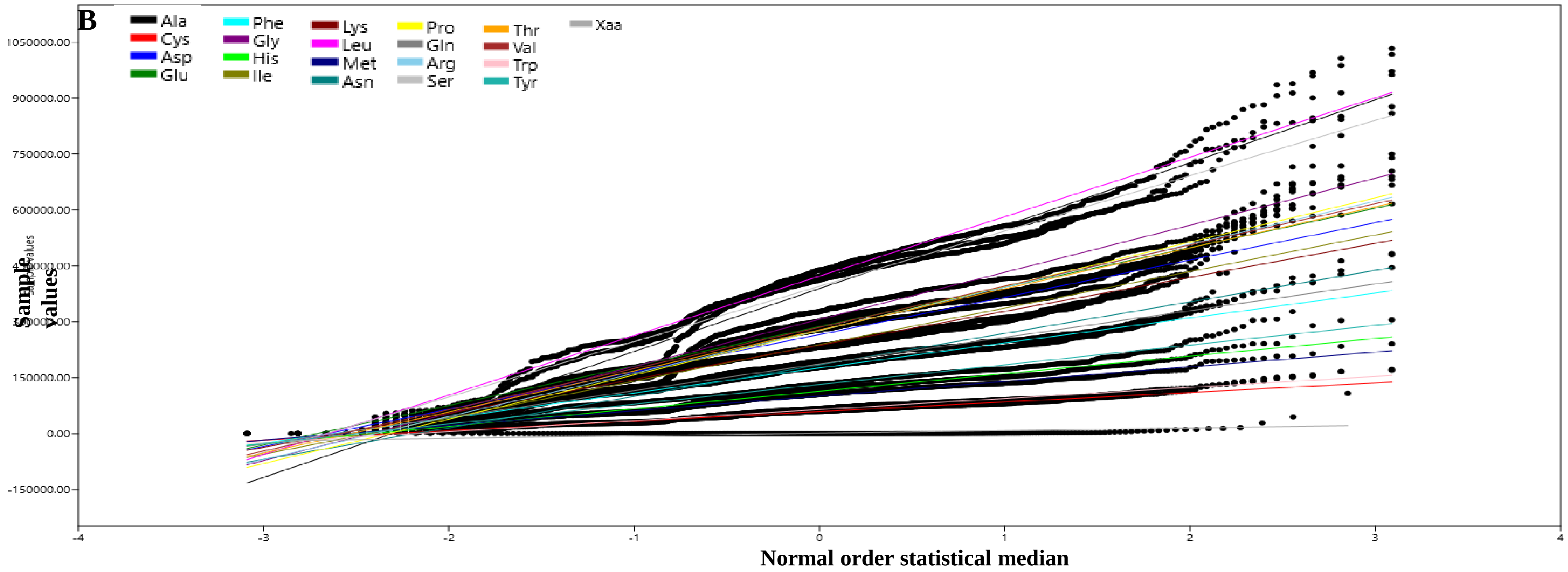

Amino acids	Correlation Coefficient
Ala	0.9772
Cys	0.984
Asp	0.990
Glu	0.987
Phe	0.988
Gly	0.983
His	0.989
Ile	0.954
Lys	0.939
Leu	0.991
Met	0.993
Asn	0.882
Pro	0.981
Gln	0.990
Arg	0.978
Ser	0.988
Thr	0.991
Val	0.990
Trp	0.990
Tyr	0.972
Xaa	0.381

Supplementary Figure 3

Normal probability distribution of fungal proteomes. (A) Normal probability distribution of acidic pI proteins (%) (correlation coefficient 0.9635, $p < 0.05$). (B) Normal probability distribution of basic pI proteins (%) (correlation coefficient 0.974, $p < 0.05$).

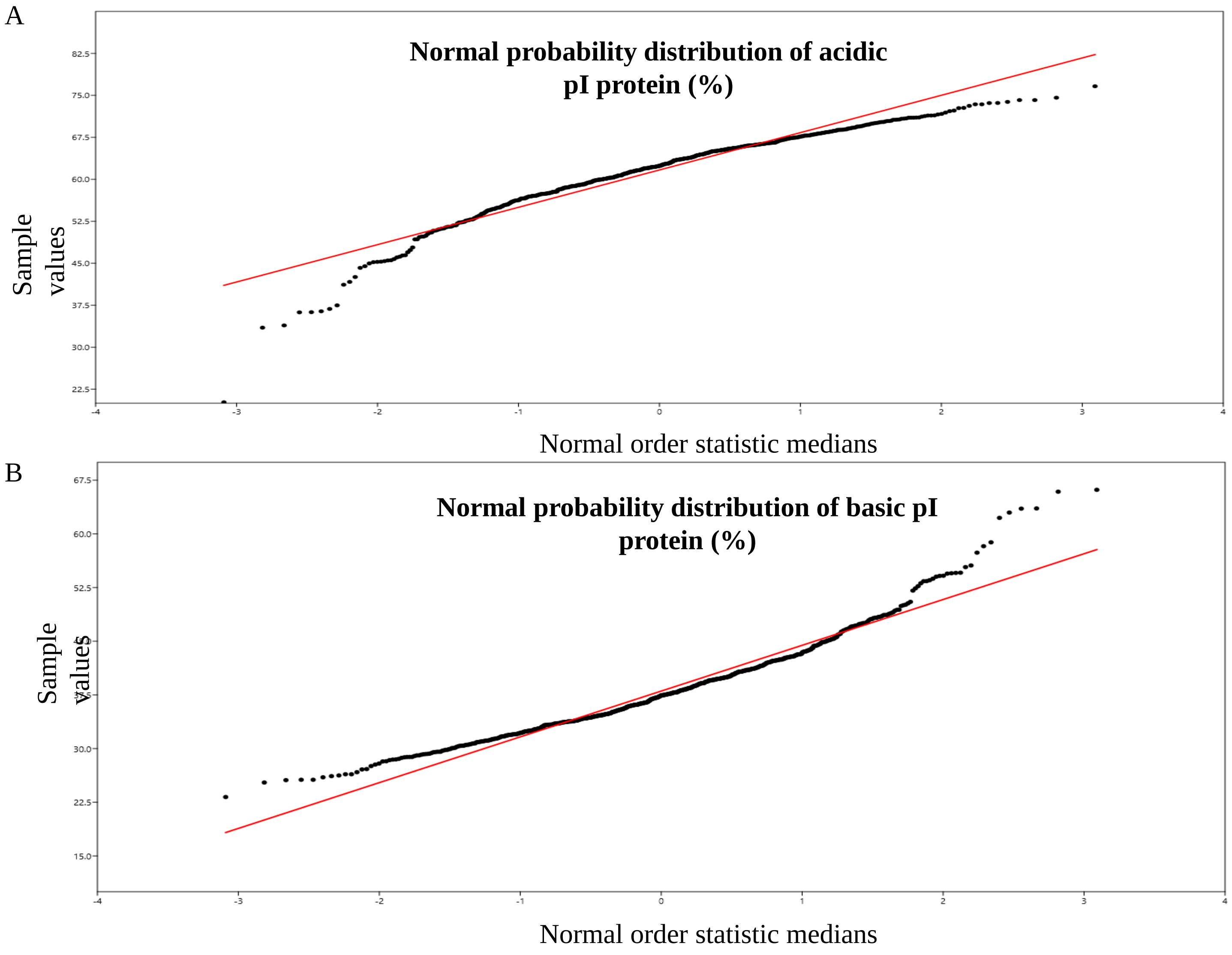

Supplementary Figure 4

Search for various amino acids in the whole proteome of *Bifiguratus adelaide*, *Gonapodya prolifera*, *Capniomyces stellatus*, *Zancudomyces culisetae*, *Smittium angustum*, *and Furculomyces boomerangus* species. The search was conducted on a linux based platform. The proteome file of individual species was downloaded from the NCBI and analyzed for the presence/absence of Sec (U) amino acid. Analysis show, there is no U amino acid in the proteome of any of the reported species. The proteome sequences of *Chlamydomonas reinhardtii* was used as reference that shows the presence of U amino acid in the search.

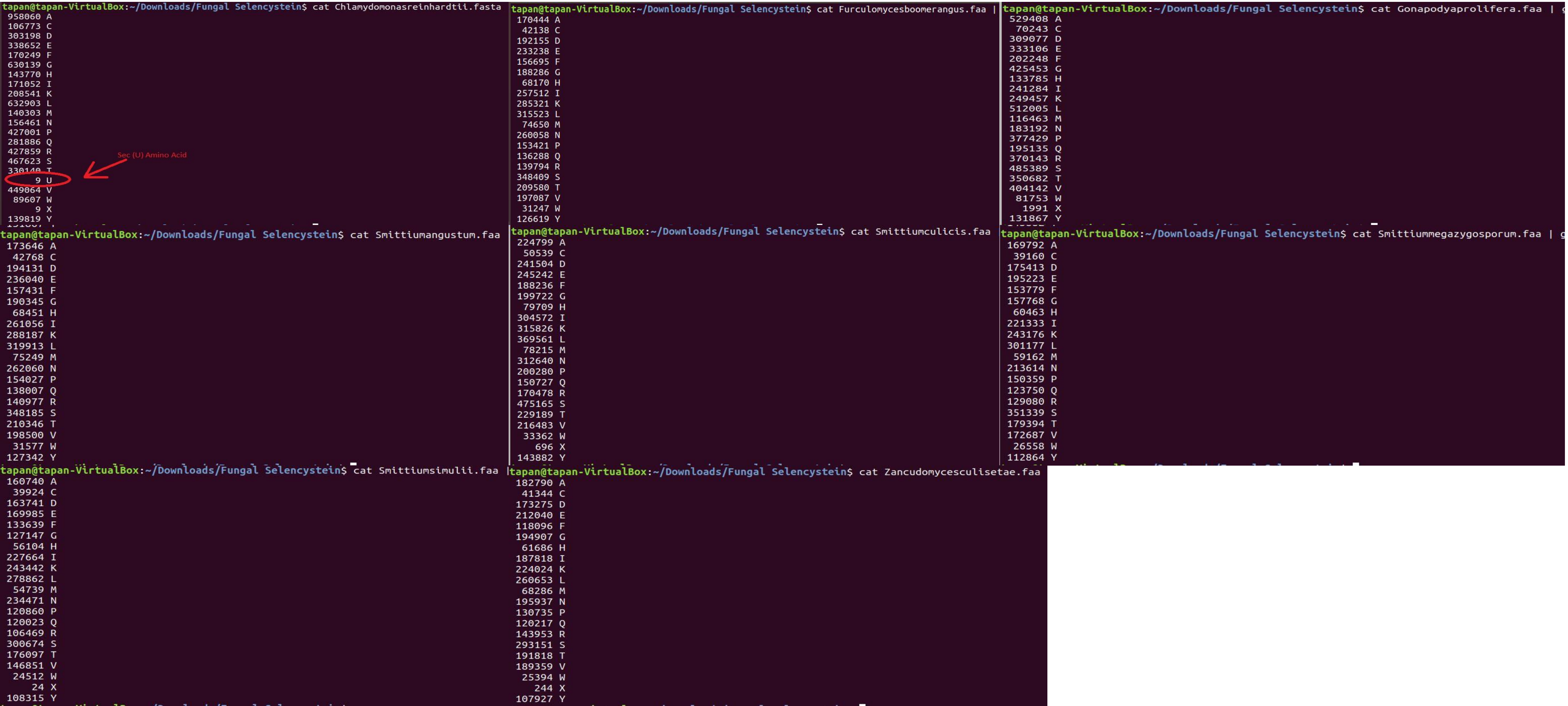

Supplement: Supplementary file 1 — Supplementary information 1. [file 41598_2021_86201_MOESM1_ESM.pdf]
